# Supplementary material for: Causal interplay between lactose intolerance and gut microbiota: a combined bidirectional Mendelian randomization and in vivo validation study
Source: Front Nutr. 2026 Jun 1;13:1803337. doi: 10.3389/fnut.2026.1803337 (PMC13265576; doi:10.3389/fnut.2026.1803337)
Supplement: Supplementary file 1 [file Data_Sheet_1.zip › supplementary materials/Forward/leave one out cross validation/ebi-a-GCST90027695.finngen_R12_E4_LACTONAS.pdf]

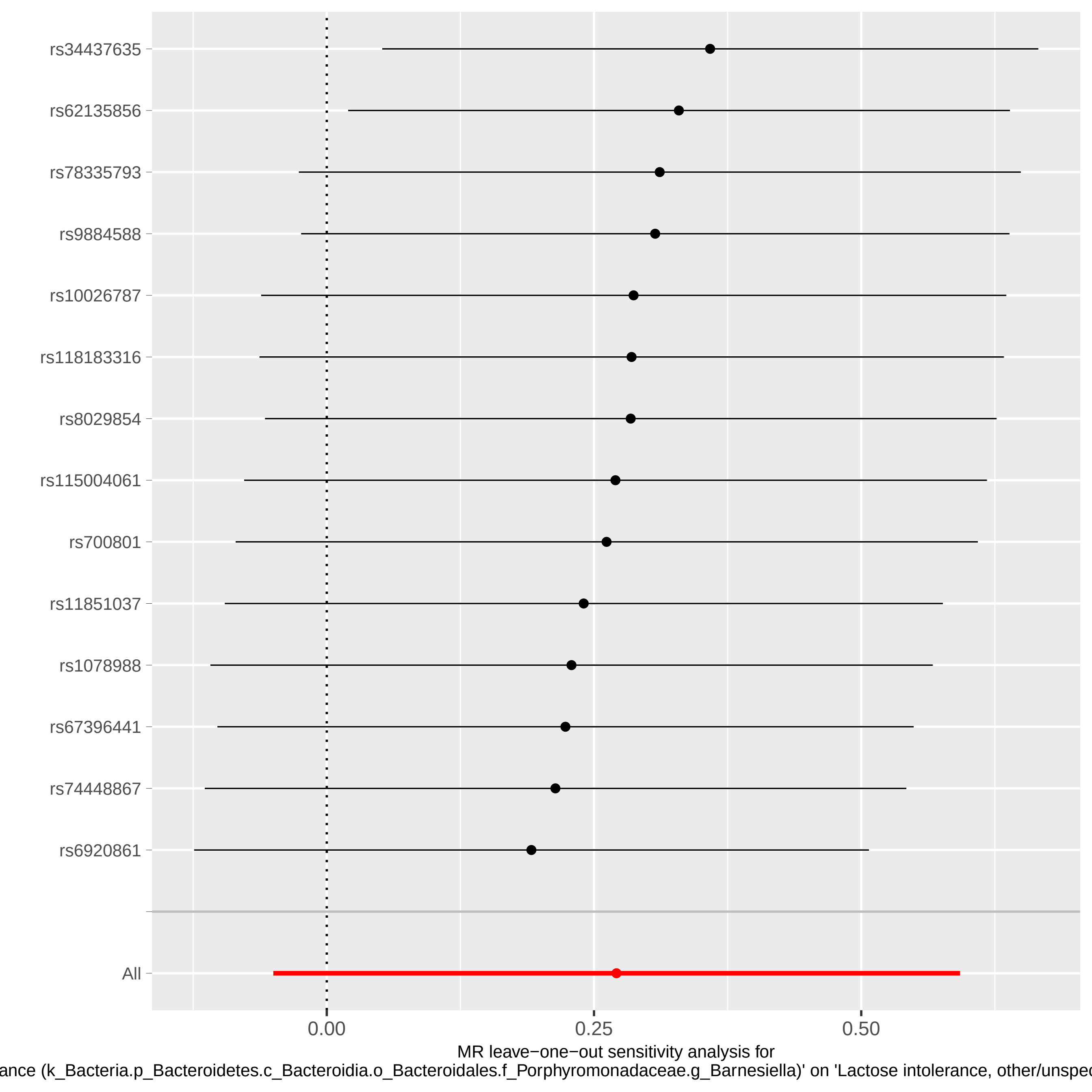

rs34437635
rs62135856
rs78335793
rs9884588
rs10026787
rs118183316
rs8029854
rs115004061
rs700801
rs11851037
rs1078988
rs67396441
rs74448867
rs6920861
All
0.00
0.25
0.50
MR leave−one−out sensitivity analysis for
ance (k_Bacteria.p_Bacteroidetes.c_Bacteroidia.o_Bacteroidales.f_Porphyromonadaceae.g_Barnesiella)' on 'Lactose intolerance, other/unspe
